# Neuronal intrinsic properties shape naturally evoked sensory inputs in the dorsal horn of the spinal cord

**DOI:** 10.3389/fncel.2013.00276

**Published:** 2013-12-25

**Authors:** Cecilia Reali, Raúl E. Russo

**Affiliations:** Neurofisiología Celular y Molecular, Instituto de Investigaciones Biológicas Clemente EstableMontevideo, Uruguay

**Keywords:** spinal cord, plateau potentials, low threshold calcium spikes, intrinsic electrophysiological properties, dorsal horn neurons, sensory information processing

## Abstract

Intrinsic electrophysiological properties arising from specific combinations of voltage-gated channels are fundamental for the performance of small neural networks in invertebrates, but their role in large-scale vertebrate circuits remains controversial. Although spinal neurons have complex intrinsic properties, some tasks produce high-conductance states that override intrinsic conductances, minimizing their contribution to network function. Because the detection and coding of somato-sensory information at early stages probably involves a relatively small number of neurons, we speculated that intrinsic electrophysiological properties are likely involved in the processing of sensory inputs by dorsal horn neurons (DHN). To test this idea, we took advantage of an integrated spinal cord–hindlimbs preparation from turtles allowing the combination of patch-clamp recordings of DHN embedded in an intact network, with accurate control of the extracellular milieu. We found that plateau potentials and low threshold spikes (LTS) -mediated by L- and T-type Ca^2+^channels, respectively- generated complex dynamics by interacting with naturally evoked synaptic potentials. Inhibitory receptive fields could be changed in sign by activation of the LTS. On the other hand, the plateau potential transformed sensory signals in the time domain by generating persistent activity triggered on and off by brief sensory inputs and windup of the response to repetitive sensory stimulation. Our findings suggest that intrinsic properties dynamically shape sensory inputs and thus represent a major building block for sensory processing by DHN. Intrinsic conductances in DHN appear to provide a mechanism for plastic phenomena such as dynamic receptive fields and sensitization to pain.

## INTRODUCTION

In neural networks, the relative weight of synaptic and intrinsic conductances varies depending on the type of neuron (Fernandez and White, 2009) as well as on the task performed (Toledo-Rodriguez et al., 2005; Berg and Hounsgaard, 2009). Although spinal neurons have complex repertoires of intrinsic properties, such as plateau potentials and low threshold spikes (LTS; Russo and Hounsgaard, 1999), their contribution to the various functions executed by spinal circuits remains controversial. Using an isolated carapace–spinal cord preparation in turtles, Alaburda et al. (2005) showed that plateau potentials in motoneurons are overridden by synaptic activity during scratch. However, inward persistent conductances in cat motoneurons innervating ankle extensor muscles are modulated by small changes in the angle of the ankle joint (Hyngstrom et al., 2007) and plateau potentials are recruited in frog motoneurons during the withdrawal reflex (Perrier and Tresch, 2005). This suggests that the involvement of intrinsic properties is highly dependent on the particular function executed by spinal circuits.

The detection and feature extraction of sensory information during the initial steps of sensory processing involve complex transformations at the cellular level. We hypothesize that as reported for some sensory modalities (Sanchez-Vives et al., 2000; Kawai and Miyachi, 2001; Oswald et al., 2004; Loewenstein et al., 2005; Tan and Borst, 2007), intrinsic properties of dorsal horn neurons (DHN) actively shape somato-sensory information carried by primary afferent fibers. To test this idea, we took advantage of an integrated spinal cord–hindlimbs preparation from turtles allowing the combination of patch-clamp recordings of DHN embedded in an intact network with accurate control of the extracellular milieu (Reali and Russo, 2005). We found that plateau potentials and LTS -mediated by L- and T-type Ca^2+^channels, respectively- generated complex dynamics by interacting with naturally evoked synaptic potentials. For example, the LTS underlined a form of plasticity of inhibitory receptive fields whereas the plateau potential transformed sensory signals in the time domain. Thus, unlike some motor tasks involving massive activation of large-scale networks, intrinsic properties have a say on the integration of sensory information performed by DHN.

## MATERIALS AND METHODS

All experimental procedures were performed in accordance with the ethical guidelines established by our local Committee for Animal Care and Research at the Instituto de Investigaciones Biológicas Clemente Estable. Every precaution was taken to minimize animal stress and the number of animals used. Data were obtained from 40 juvenile turtles (*Trachemys dorbignyi*; 5–7 cm carapace length). The animals were maintained in temperate aquaria (24–26°C) under natural illumination.

### INTEGRATED PREPARATION

The procedures to obtain the preparation are described in detail elsewhere (Reali and Russo, 2005). Briefly, turtles rendered torpid by hypothermia induced by immersion in crushed ice for 1.5–2 h (Melby and Altman, 1974; Alaburda et al., 2005) were decapitated and the blood was removed by intraventricular perfusion with Ringer solution (6°C) of the following composition (in millimolar): 96.5 NaCl, 2.6 KCl, 31.5 NaHCO_3_, 4 CaCl_2_, 2 MgCl_2_, and 10 glucose. The solution was saturated with 5% CO_2_ and 95% O_2_ to attain pH 7.6. The animals were curarized (50–40 mg kg^-^^1^, I.M.) to avoid reflex responses to sensory stimulation. The lumbo-sacral spinal cord was exposed on its dorsal side by remotion of a strip of carapace, and a chamber was formed by fixing two blocks of agar along the cord. The posterior half of the body was then glued to a platform for recording and stimulation. The spinal cord was continuously superfused with Ringer solution at a rate of 1 ml min^-^^1^. In some experiments, NiCl_2_ (200–900 μM), CsCl_2_ (1 mM), and nifedipine (10–50 μM, Sigma) were added to the Ringer solution. At least 15 min elapsed before data collection after a change in composition of the superfusate. All experiments were performed at room temperature (20–22°C).

### ELECTROPHYSIOLOGICAL RECORDINGS AND STIMULATION

Patch-clamp whole-cell recordings were made blindly in the lumbar enlargement at depths of 150–500 μm from the dorsal surface of the cord. The electrodes (7–20 MΩ) were pulled from thick wall glass tube (A-M Systems, Carlsborg, WA, USA) with a Flaming–Brown P-87 puller (Sutter Instruments, Co., USA) and filled with the following solution (in millimolar): 122 K-gluconate, 5 Na_2_-ATP, 2.5 MgCl_2_, 0.0003 CaCl_2_, 5.6 Mg-gluconate, 5 K-Hepes, 5 H-Hepes. In some experiments, biocytin (10 mM) was also added to the patch solution. Recordings were performed in the current clamp mode with an AxoClamp-2B amplifier (Axon Instruments, Union City, CA, USA) driven by a programmable stimulator (Master-8; A.M.P.I., Israel). Data were filtered (DC-5 KHz), digitized (20 kHz sampling rate), and stored in a personal computer for offline analysis.

The passive and active properties of DHN were characterized by applying current pulses lasting from 500 ms to 5 s at different levels of holding current. Action potential amplitudes were measured from peak to peak, input resistances determined in the linear region of the voltage-current relationship and liquid junction potentials (-14.6 mV) corrected off-line (Barry and Diamond, 1970). Numerical values are expressed as mean ± SEM.

After the electrophysiological characterization of the recorded cell, the receptive field was studied by applying mechanical stimuli in the dorsal surface of the ipsilateral hindlimb. To map the receptive field, stimuli were applied in 3 mm steps and repeated three times at each location. Innocuous mechanical stimuli were produced by means of a fine artist brush and pinprick stimuli with the tip of a fine tweezers. To ensure the same level of natural stimulation when performing pharmacology, we applied vibratory stimuli to the skin with a blunt probe (0.6 mm diameter) or a sharp tip attached to the cone of a loudspeaker. A wave generator (Hewlett Packard 3312A) was used to drive the loudspeaker to produce sinusoidal stimuli of 60–70 Hz. A homemade movement detector based on an infrared optocoupler was used to measure the displacement of the loudspeaker cone (Reali and Russo, 2005).

## RESULTS

### RESPONSE PROPERTIES OF BURSTING NEURONS TO NATURAL STIMULATION

**FIGURE 1:**
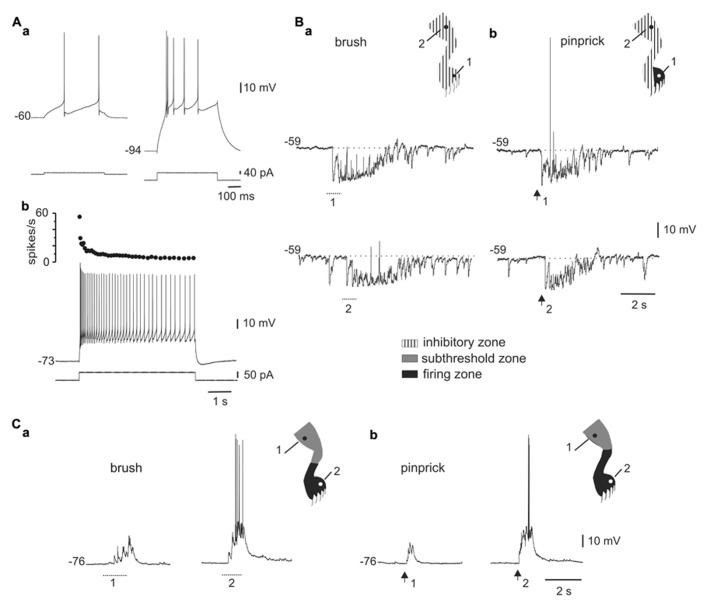
**Responses to mechanical stimulation in cells with LTS. (A)** A depolarizing current pulse produced low-frequency tonic firing when applied at rest [**(a)**, left] and a high-frequency burst when the cell was hyperpolarized with bias current [**(a)**, right]. Application of a long-lasting current pulse (5 s) shows that the initial high-frequency burst is followed by sustained tonic firing **(b)**. **(B)** Responses of the cell shown in A to brush **(a)** and pinprick **(b)** applied in two different zones of the ipsilateral hindleg (1 and 2, dots in the cartoons). **(C)** Responses of a different bursting cell to brush **(a)** and pinprick **(b)**. The corresponding receptive fields are shown in the insets. In this and subsequent figures, dotted lines and arrows underneath the traces indicate the time of application of the brush and pinprick stimuli, respectively. (**Aa–Ab)** and **(Ca–Cb)** from the same cell.

**FIGURE 2:**
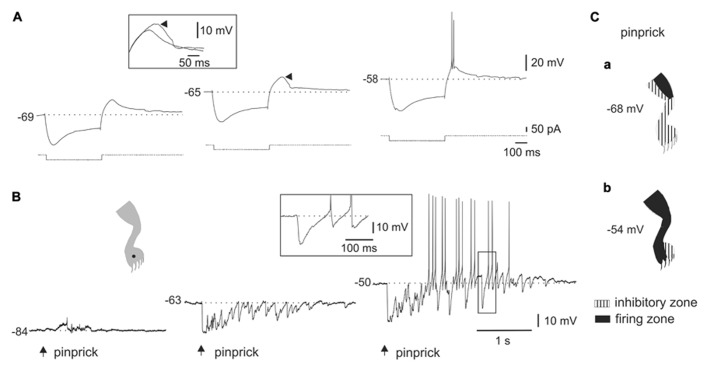
**Interaction between the LTS and synaptic activity elicited by natural stimulation. (A)** A hyperpolarizing current pulse applied at different levels of bias current. Notice that the rebound response grew as the membrane potential was depolarized to generate a burst of action potentials (-58 mV). The inset shows the superimposed rebounds generated at -69 and -65 mV (arrowhead). **(B)** In the cell shown in **(A)**, a barrage of IPSPs was generated when pinprick was applied to a spot within the receptive field (dot in cartoon). As the membrane potential was depolarized, the IPSPs generated action potentials after some delay. The inset shows that spiking resulted from post-inhibitory rebounds. **(C)** Cartoons showing the receptive field of a DHN with a naturally induced response similar to that shown in B, at two different membrane potentials. The firing zone at rest [**(a)**, -68 mV] was smaller than at depolarized membrane potentials [**(b)**, -54 mV] as the LTS interacted with IPSPs.

As previously described in slices (Ryu and Randic, 1990; Russo and Hounsgaard, 1996b), we found that some DHN in the integrated spinal cord-hindlimbs preparation showed burst firing when depolarized from hyperpolarized membrane potentials (**Figure 1Aa**) or at the offset of hyperpolarizing current pulses (**Figure 2A**). As shown in **Figure 1**, the same absolute level of current during the pulse generated a mild response at rest (**Figure 1Aa**, left trace) but a strong burst of action potentials when bias current hyperpolarized the cell (**Figure 1Aa**, right trace), suggesting the activation of an LTS (Jahnsen and Llinás, 1984). Long-lasting current pulses in bursting neurons generated an initial high frequency of action potentials that subsided over many seconds to end in tonic firing (**Figure 1Ab**). Bursting cells (*n* = 50) were found in relatively superficial layers of the dorsal horn (78%, 150–300 μm below the surface), had action potential amplitudes of 74.7 ± 1.6 mV (*n* = 47), input resistances of 1.3 ± 0.1 GΩ (*n* = 48), and resting membrane potentials of -70.0 ± 1.4 mV (*n* = 41).

**FIGURE 3:**
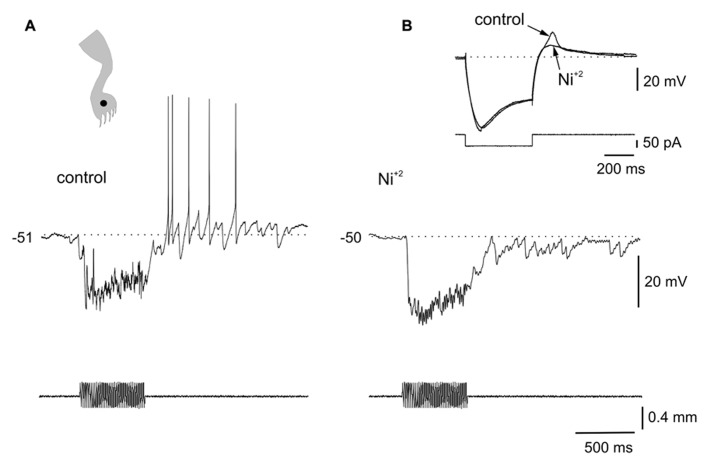
**Ionic mechanisms of the interaction between LTS and sensory inputs. (A)** Sinusoidal (70 Hz) mechanical stimulation applied on the ipsilateral leg (dot in cartoon) produced a strong hyperpolarization followed by spiking generated by post-inhibitory rebounds. **(B)** In the presence of Ni^2^^+^ (300 μM), the same stimulus produced a strong inhibition but delayed spike firing was eliminated. The inset in **(B)** shows that the LTS induced by a hyperpolarizing current pulse was strongly reduced by Ni^2^^+^. All data from the same cell.

The responses to brush (**Figure 1Ba**) or pinprick (**Figure 1Bb**) of the skin in most bursting neurons (33 of 41) were dominated by a barrage of inhibitory post-synaptic potentials (IPSPs) intermingled with a few excitatory post-synaptic potentials (EPSPs). At resting membrane potentials, IPSPs were identified as rapid hyperpolarizing deflections followed by a slower relaxation, whereas EPSPs were conversely recognized as fast depolarizing events. In addition, as the membrane was hyperpolarized with holding current, IPSPs decreased in amplitude (see **Figure 2B**) -reversing close to the Cl^-^ equilibrium potential (-78.3 mV at 20°C) – whereas EPSPs were decreased in amplitude by depolarization. In 19 of 29 cells, the inhibitory receptive fields were large, comprising the whole ipsilateral leg and had a small firing zone (**Figure 1Bb**, upper trace and cartoon in inset). The hyperpolarizing response elicited by brief stimulation within the inhibitory receptive field could last up to 2 s (see **Figures 1B, 2B**, and **3**) and may be due to repetitive firing of inhibitory interneurons. The remaining neurons with LTS (8 out of 41) showed responses to brush (**Figure 1Ca**) or pinprick (**Figure 1Cb**) composed mostly by EPSPs. In these cells, the excitatory receptive fields had a firing zone surrounded by a subthreshold zone (**Figure 1C**, cartoons in insets).

### INTERACTIONS OF THE LTS WITH NATURALLY EVOKED SENSORY INPUTS

Although previous studies in slices showed that LTS could interact with synaptic inputs elicited by stimulation of dorsal roots (Russo and Hounsgaard, 1996b), it is not clear whether LTS could shape the output of DHN when driven by meaningful sensory inputs. We thus analyzed the interactions of naturally evoked sensory inputs with the intrinsic properties of bursting cells. **Figure 2** shows the responses of a bursting cell to hyperpolarizing current pulses (**Figure 2A**) and to natural stimulation (**Figure 2B**) at different membrane potentials. The presence of a sag during the pulse suggested that the post-inhibitory rebound was partly accounted for by the activation of a time-dependent anomalous rectification (**Figure 2A**, left trace). However, a substantial component of the response was mediated by the activation of an LTS since the rebound grew (**Figure 2A**, middle trace, arrowhead) to become a burst of spikes with progressive application of depolarizing holding current (**Figure 2A**, right trace). The inset in **Figure 2A** shows the superimposed rebound responses at -69 and -65 mV. At hyperpolarized and resting membrane potentials (**Figure 2B**, left trace and middle trace, respectively), the cell responded to pinprick with a strong and long-lasting inhibition. However, when the cell was held at depolarized membrane potentials (**Figure 2B**, right trace), pinprick on the same spot of the skin (**Figure 2B**, dot in cartoon) generated an early barrage of IPSPs followed by spike firing at the end of the response. Notice that spiking did not arise from EPSPs but from the rebound produced by individual IPSPs as shown on a faster time scale in the boxed inset. **Figure 2C** shows the effects of changing the membrane potential on the characteristics of the receptive field. The large inhibitory receptive field measured at rest (**Figure 2Ca**, -68 mV) changed to an extended firing zone when the bursting neuron was depolarized (**Figure 2Cb**, -54 mV).

The voltage dependence of the responses induced by natural stimulation of the receptive field suggests that the delayed excitation following inhibition was mediated by the activation of T-type Ca^2^^+^ channels. To confirm that the delayed excitation was due to the interaction of IPSPs and the LTS we used Ni^2^^+^ (200–900 μM) as a T-type Ca^2^^+^ channel blocker. In all cases (*n* = 6), the rebound responses were reduced in the presence of Ni^2^^+^. **Figure 3** shows that a delayed excitation in response to sinusoidal (70 Hz) stimulation with a sharp probe (**Figure 3A**) disappeared when Ni^2^^+^ was added to the bath (300 μM, **Figure 3B**). Notice, however, that the inhibition induced by the same stimulus in the presence of Ni^2^^+^ was even larger than that of control, suggesting the blockade of the delayed excitation is because of the antagonism of post-synaptic T-type Ca^2^^+^ channels. In line with this, Ni^2^^+^ selectively blocked the T-type Ca^2^^+^ channel component of the LTS generated at the offset of a hyperpolarizing current pulse (**Figure 3B**, inset, *n* = 6). The contribution of the time-dependent anomalous rectification to rebound responses induced by natural stimulation in bursting DHN seems to be small because Cs^2^^+^ (1 mM, *n* = 3) did not prevent delayed excitation (data not shown).

### RESPONSES OF DHN WITH PLATEAU PROPERTIES TO NATURAL STIMULATION

**FIGURE 4:**
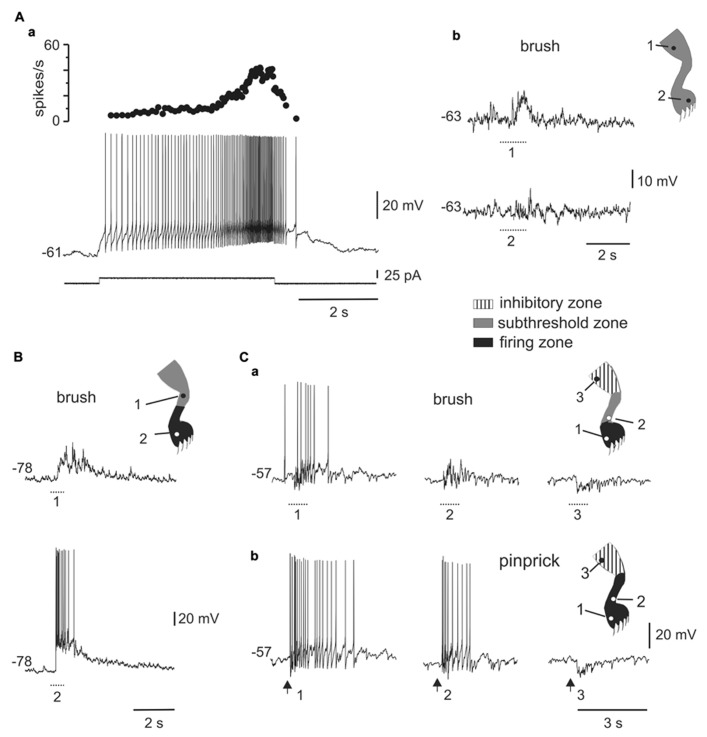
**Responses of plateau-generating neurons to sensory stimulation. (A)** Incrementing firing frequency and after-discharge generated by a depolarising current pulse **(a)**. In the same cell, brushing the skin within different zones [**(b)**, 1 and 2] of the ipsilateral leg generated sub-threshold synaptic responses. **(B)** Most plateau neurons had an excitatory receptive field with a sub-threshold zone (1) and a firing zone (2). **(C)** Plateau-generating neuron that had a receptive field with excitatory (1, 2) and inhibitory (3) zones in response to innocuous **(a)** as well as noxious **(b)** mechanical stimuli. Note that the firing zone was larger to pinprick **(b)** than to brush **(a)**.

A second population of cells (*n* = 51) responded with incrementing firing frequency of action potentials and after-discharges in response to long-lasting depolarizing current pulses (**Figure 4Aa**). Neurons with plateau potentials localized more deeply in the dorsal horn (80%, 250 μm to 500 below the surface), had spike amplitudes of 79.6 ± 1.3 mV (*n* = 51), input resistances of 1.2 ± 0.1 GΩ (*n* = 51), and resting membrane potentials of -58.5 ± 1.3 mV (*n* = 47).

All plateau neurons responded to mechanical stimuli applied to the ipsilateral hindlimb and had large receptive fields. The majority of cells (31 out of 35) were wide dynamic range (WDR) neurons since they responded to brush and pinprick of the skin (**Figure 4C**). In 11 of 47 cells, the response to skin brush consisted of subthreshold EPSPs mixed with some IPSPs (**Figure 4Ab**), whereas in the remaining cells a firing zone within the receptive field was observed (**Figures 4B,C**). The responses were complex with concurrent activation of excitation and inhibition. As the stimulus moved away from the firing zone, the inhibitory component of the response became stronger, to turn in some cells (6 out of 36) into a net hyperpolarization that defined an inhibitory zone within the receptive field (**Figure 4C**).

### PLATEAU POTENTIALS INTERACT WITH NATURALLY EVOKED SENSORY INPUTS

**FIGURE 5:**
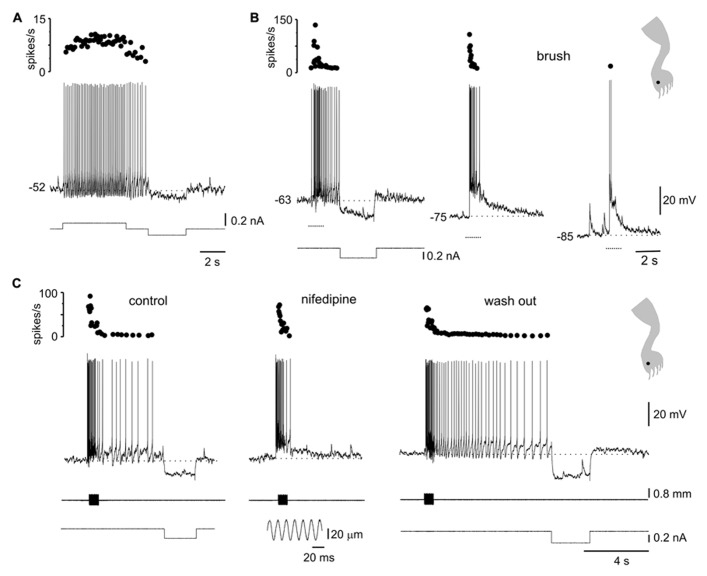
**Plateau potentials in DHN shape naturally evoked sensory inputs. (A)** Incrementing firing frequency due to activation of a plateau potential. The stimulus was followed by an after-discharge terminated by a hyperpolarizing current pulse. **(B)** Response to brush on a spot of the receptive field (inset) at different membrane potentials. **(C)** A sinusoidal (60 Hz, lower inset) mechanical stimulation with a fine probe on a spot within the receptive field (cartoon in inset on the right) activated the plateau potential as suggested by the prolonged after-discharge terminated by a hyperpolarizing current pulse (control). Nifedipine (40 μM) spared the earliest component of the response induced by natural stimulation but blocked the prolonged after-discharge which reappeared after wash out. All data from the same cell.

Plateau properties have been implied in the generation of persistent activity in sensory systems (Lo and Erzurumlu, 2002; Matsumoto et al., 2009) and during some motor tasks (Major and Tank, 2004; Perrier and Tresch, 2005; Hyngstrom et al., 2007). As previously described in slices (Russo and Hounsgaard, 1994, 1996a; Morisset and Nagy, 1998), a depolarizing current pulse in DHN with plateau properties can elicit persistent firing that can be turned off by transient hyperpolarization (**Figure 5A**). In these cells, persistent activity could also be triggered by natural stimulation within the excitatory receptive field (**Figure 5B**, -63 mV). Notice that although the persistent response was synaptically induced, it could be terminated by a hyperpolarizing current pulse. Hyperpolarizing the membrane potential with bias current reduced the number of spikes and the overall duration of the response (**Figure 5B**, -75 and -85 mV).

The voltage-dependence of the responses to natural stimulation suggests that L-type Ca^2^^+^ channels in the post-synaptic membrane add substantially to the responses induced by brief stimulation of the receptive field. Indeed, the persistent activity induced by mechanical stimulation within the excitatory receptive field was reversibly eliminated by nifedipine (**Figure 5C**; 20–50 μM, *n* = 5). Notice that the early, synaptically driven component of the response was unaffected by nifedipine.

**FIGURE 6:**
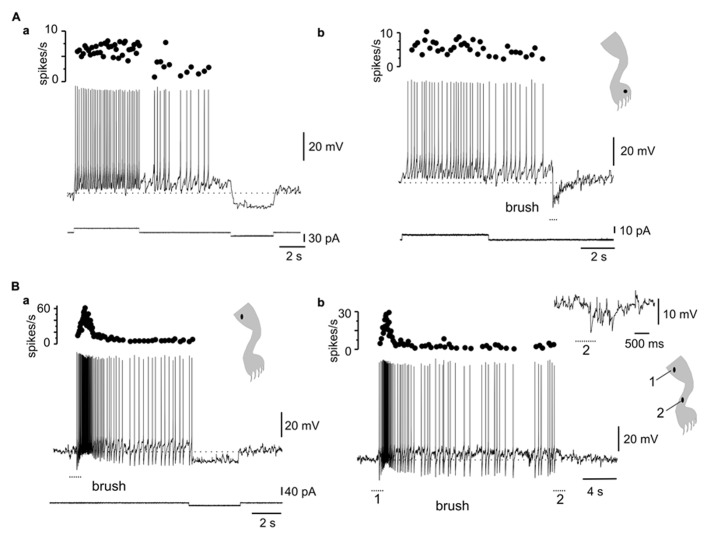
**Complex dynamics between plateau properties and stimulation in different receptive field spots. (A)** The after-discharge produced by depolarizing current pulses could be turned off by a hyperpolarizing current pulse **(a)** or by brief brush within the inhibitory receptive field **(b)**. **(B)** Gentle mechanical stimulation induced a strong response with incrementing firing frequency followed by an after-discharge terminated by a mild hyperpolarizing current pulse **(a)**. A similar behavior could be produced by alternate stimulation on spots within the excitatory (1) and inhibitory (2) receptive fields **(b)**. The inset in **(b)** shows the response to brush on a spot within the inhibitory receptive field. **(A,B)** From two different cells.

Stimulation within different zones of the receptive fields of DHN with plateau properties generated complex dynamics. For example, the after-discharges mediated by L-type Ca^2^^+^ channels (**Figure 6Aa**) could be terminated by transient stimulation of the inhibitory zone of the receptive field (**Figure 6Ab**, 5 out of 5 cells). In fact, bistability could be produced by alternate stimulation within the excitatory and inhibitory zones of the receptive field (**Figure 6B**, 4 out of 4 cells).

**FIGURE 7:**
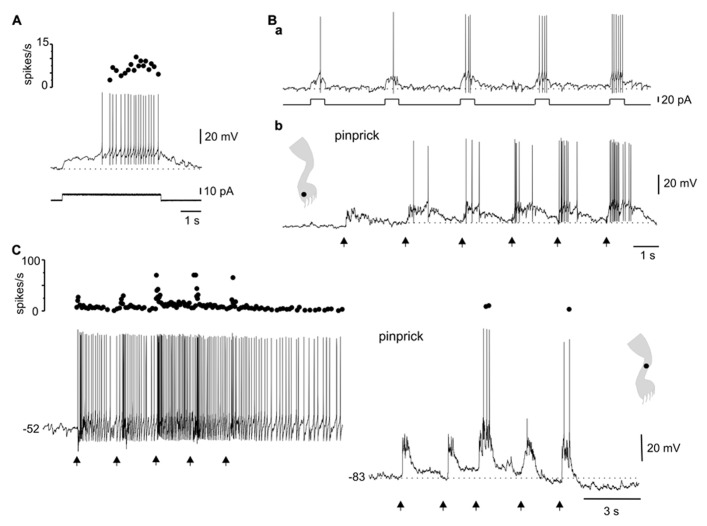
**Integration of sensory information in the time domain by plateau potential generating neurons. (A)** A depolarizing current pulse produced incrementing firing frequency followed by a depolarizing after-potential. **(B)** Windup of the response to repeated current pulses **(a)** as well as to repeated pinprick on a spot (dot in cartoon) of the excitatory receptive field **(b)**. **(C)** Windup of the response to repeated pinprick at rest (-52 mV, left trace). The windup of the response disappeared at hyperpolarized membrane potentials (-83 mV, right trace). **(A,B)** From the same cell.

**FIGURE 8:**
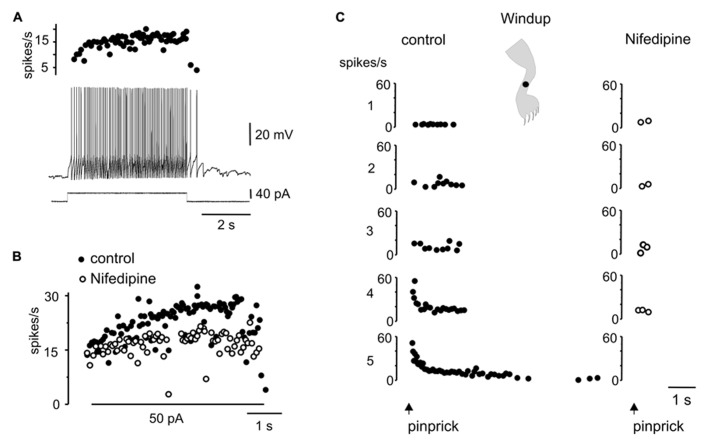
**Windup to repeated sensory stimulation is eliminated by blockade of L-type Ca^2+^ channel. (A)** Response to a depolarizing current pulse in a plateau potential generating cell. **(B)** Instantaneous firing frequency in response to 50 pA current pulse before (control) and after addition of nifedipine (50 μM). **(C)** Repeated noxious mechanical stimulation applied in the ipsilateral leg produced a windup of the response in control conditions but not in the presence of nifedipine (50 μM). All data from the same cell.

Another interesting dynamic generated by the plateau potential occurred within the time domain. As described previously in slices of the turtle (Russo and Hounsgaard, 1994, 1996a) and rat (Morisset and Nagy, 1998, 2000) spinal cords, the repetition of a mild depolarizing current pulse in DHN with plateau properties (**Figure 7A**) induced a “windup” of the response (**Figure 7Ba**, 21 out of 29 cells). The facilitation of the response could be explained by the “warm-up” of L-type Ca^2^^+^ channels as there was no cumulative depolarization between stimuli (**Figure 7Ba**). In 15 out of 19 cells, the wind up produced with current pulses could also be generated by natural stimulation of the skin. For example, in the cell shown in **Figure 7Ba**, repetitive pinprick within the subthreshold zone of the receptive field produced spike firing “windup” (**Figure 7Bb**) similar to that induced with current pulses. The offset of windup varied widely among plateau-generating cells, ranging from about 5 s to hundreds of seconds when persistent firing occurred. **Figure 7C** shows a plateau neuron in which repetitive pinprick applied in the firing zone of the receptive field induced “windup” of the response followed by persistent firing at the resting membrane potential (**Figure 7C**, -52 mV). The same stimulation protocol applied at hyperpolarized membrane potentials (**Figure 7C**, -83 mV) showed that the synaptic drive induced by natural stimulation did not increase with repetition, suggesting that windup to pinprick was mediated by the intrinsic properties of DHN. To confirm this interpretation, we tested the effect of L-type Ca^2^^+^ channel blockade with nifedipine on windup generated by pinprick of the skin. Nifedipine (20–50 μM, *n* = 5) reduced the incrementing firing frequency during a long-lasting depolarizing current step (**Figures 8A,B**) and in the same cell wiped out the windup of the response to repetitive pinprick (**Figure 8C**). Collectively, our data show that the windup of the response is produced by the integration of inputs by the L-type Ca^2^^+^ channels over a slow time frame and not by a progressive increase in synaptic weight.

## DISCUSSION

Intrinsic properties represent a major building block in small-scale networks of invertebrates (Getting, 1989; Marder and Calabrese, 1996). Although neurons in vertebrates also have complex intrinsic dynamics (Llinás, 1988), their relevance in large-scale networks has been questioned (Alaburda et al., 2005; Berg et al., 2007; Kolind et al., 2012). We show here that LTS and plateau potentials dynamically shaped naturally evoked sensory inputs in DHN immersed within an intact spinal network. The interaction of intrinsic properties and synaptic potentials occurred within voltage and time windows defined by the properties of T-type and L-type Ca^2+^channels.

### LTS IN DHN: EXCITED BY INHIBITION

Most DHN with LTS responded to natural stimulation with a robust barrage of IPSPs resulting in receptive fields with large inhibitory components. Interestingly, the interaction of the synaptic potentials and the LTS within a narrow range of membrane potentials produced post-inhibitory rebounds that paradoxically transformed an inhibitory input into a delayed excitatory output. Rebound excitation after inhibition by noxious stimuli in the hindpaw has been reported in a subset of DHN recorded extracellularly in rats (McGaraughty and Henry, 1997) and in cat spinoreticular neurons after powerful IPSPs elicited by sciatic nerve stimulation (Sahara et al., 1990). Thus, rebound excitation seems a general computational element during the analysis of somato-sensory information in the spinal cord. The temporal sequence of inhibition-excitation has been generally assumed to arise from the logic of circuitry (Large and Crawford, 2002). Neurons with rebound responses to hyperpolarizing current pulses and inhibitory receptive fields were found in a hemisected spinal cord-hindlimb preparation of the neonatal rat (Lopez-Garcia and King, 1994) and *in vivo* in superficial DHN of the mouse (Graham et al., 2004), although in these studies the possible interaction with LTS was not explored. Our study shows that depending on the recent history of DHN (e.g., the actual membrane potential at which inhibition occurs) rebound excitation can be fully accounted for by the interaction of sensory inputs and the properties of the post-synaptic membrane.

The function of the intrinsic delayed excitation produced by the LTS in DHN is puzzling. In the auditory system, post-inhibitory rebound excitation has been implied in temporal computation of auditory signals (Large and Crawford, 2002). Indeed, rebound excitation in a subset of neurons in the superior paraolivary nucleus and the inferior colliculus is produced by intrinsic mechanisms activated by hyperpolarization induced by acoustic stimulation (Felix et al., 2011; Kasai et al., 2012). It is then possible, that the interaction between sensory-induced inhibition and the LTS in DHN similarly encode temporal features of the stimulus.

Delayed excitation is one of the functional building blocks in neuronal networks and has been proposed as an intrinsic mechanism for integration of excitatory inputs (Getting, 1989; Turrigiano et al., 1996). As proposed for the slow post-inhibitory rebound in lateral pyloric neurons of the stomatogastric ganglion (Goaillard et al., 2010), the delayed excitation induced by naturally evoked inhibition in DHN could be an intrinsic mechanism for integration of inhibitory inputs. Post-inhibitory rebounds have been also implied in the production of different forms of rhythmic motor patterns (Getting, 1989). In the neonatal rat, a post-inhibitory rebound mediated by low voltage-gated Ca^2^^+^ channels is engaged in spinal motoneurons during locomotion (Bertrand and Cazalets, 1998). In addition, a subset of interneurons, in the turtle spinal cord are rhythmically hyperpolarized during fictive scratch and swimming (Berkowitz, 2007, 2010). Although we never observed rhythmic activity in our study, DHN with LTS may be elements of the pre-motor network devoted to early stages of sensory-motor integration. Indeed, DHN with LTS are small interneurons with an axon bearing profuse collaterals in the dorsal horn (Russo and Hounsgaard, 1996b; Reali and Russo, 2005).

### PLATEAU POTENTIALS INTEGRATE SOMATOSENSORY INPUTS IN A SLOW TIME SCALE

In the somatosensory system, prolonged after-discharges in WDR neurons have been related to pain mechanisms (Wall and Melzack, 1989). Noxious stimuli applied to the skin in mammals produce prolonged post-discharges (Price et al., 1971; Coghill et al., 1993; Reali et al., 2011) and psychophysical studies showed that pain perception greatly outlasts the stimulus (Coghill et al., 1993). Although local recurrent networks are a widely accepted theory to explain persistent neuronal activity (Major and Tank, 2004), anatomical evidence supporting this kind of circuits in the spinal cord is lacking. We demonstrate here that persistent firing induced by natural stimulation can be accounted for by the activation of a plateau potential mediated by L-type Ca^2^^+^ channels. The time- and voltage-dependent facilitation of the plateau potential was also responsible for the windup of the response, a form of short-term synaptic plasticity induced by repetitive noxious stimulation (Mendell, 1966). Because the windup is considered an intermediate step in the development and maintenance of a central sensitization to pain (MacMahon et al., 1993; Urban et al., 1994), the plateau potential could be a key element of the mechanisms that generate long-lasting phenomena such as hyperalgesia. In line with this, *in vivo* extracellular recordings in the rat showed that the windup of both the response of deep DHN and the nociceptive flexion reflex were sensitive to L-type Ca^2+^channel blockers (Fossat et al., 2007). In addition, allodynia is reversed *in vivo* after blocking the expression of Ca_v_1.2 channels (Fossat et al., 2010). Remarkably, Cav1.2 mRNA is increased in a neuropathic pain model (Fossat et al., 2010), a change probably related to the increased proportion of DHN able to display plateau potentials (Reali et al., 2011).

The plateau potential could also play an important part in the processing of non-noxious sensory information as in some cases it was activated by innocuous stimuli. In mammals, some neurons display prolonged after-discharges in response to innocuous stimulation (Price et al., 1978) and in humans, innocuous stimuli can generate prolonged post-stimulus sensations (Price et al., 1978). It has been suggested that persistent neural activity to brief stimuli represents a universal form of working memory mechanism (Marder et al., 1996; Major and Tank, 2004). Thus, the plateau potential in DHN may work as an intrinsic mnemonic mechanism underlying prolonged post-stimulus sensory perceptions. The intrinsic dynamics provided by L-type Ca^2+^channels may also be a key component of sensory-motor integration at the spinal cord level. Currie and Stein (1990) proposed that the prolonged post-discharges and temporal facilitation to repetitive stimulation at low frequencies (0.2 Hz) in a set of interneurons is related to the residual excitability after the end of scratching. In fact, recent findings suggest that increased activity of the pre-motor network contributes to scratch initiation (Guzulaitis et al., 2013). DHN with plateau properties are likely components of pre-motor networks as they have axon collaterals in the ventral horn (Russo and Hounsgaard, 1996a). Therefore, after-discharges and windup induced by L-type Ca^2^^+^ channels may contribute to short-term storage and accumulation of sensory information required for the execution of motor tasks.

### PLASTICITY OF THE RECEPTIVE FIELDS MEDIATED BY INTRINSIC PROPERTIES

The receptive field of sensory neurons is not a rigid attribute but changes dynamically to adjust to changing demands (Devor and Wall, 1981; Cook et al., 1987). For example, receptive fields in WDR neurons of monkeys expand or contract depending on the attentional state of the animal (Dubner et al., 1981; Hayes et al., 1981; Hoffman et al., 1981). The mechanisms underlying the plasticity of receptive fields are poorly understood and have been related mainly to dynamic adjustments of synaptic strength. Our results suggest that both LTS and plateau potentials may mediate some forms of receptive field plasticity on a relatively fast time scale. The shift from an inhibitory to an excitatory receptive field depended on whether the membrane potential of the post-synaptic cell allowed the interaction between the IPSPs and the LTS and not on plasticity of the synaptic input itself. The dynamics of plateau potentials in DHN could also be an intrinsic mechanism for plasticity of receptive fields in the domain of time. As we showed here, frequency-dependent facilitation of L-type Ca^2^^+^ channels enabled previously sub-threshold EPSPs to generate spike firing, expanding the firing zone with the contraction of the low-probability firing fringe of the receptive field, a form of short-term plasticity of excitatory receptive fields. In addition to an excitatory receptive field, WDR neurons in mammals frequently exhibit an inhibitory receptive field (see Willis and Coggeshall, 1991). In some DHN of the cat (Cadden, 1993) and rats (Reali et al., 2011) prolonged after-discharges could be terminated by a transient stimulus within the inhibitory receptive field and turned on again by stimulation of the excitatory zone of the receptive field. The deactivation of plateau potentials by inhibitory synaptic inputs could be the mechanism generating this phenomenon and could contribute to the hypoalgesic influence elicited by stimuli applied outside the excitatory receptive field (Melzack, 1975).

Because the receptive field plasticity mediated by both LTS and plateau potentials was exquisitely sensitive to the membrane potential at which naturally evoked synaptic inputs occurred, the modulation of membrane potential by monoamines released from the brainstem (Garraway and Hochman, 2001; Sonohata et al., 2004; Yoshimura and Furue, 2006), and GABA and glycine released from the medulla (Kato et al., 2006) may be a suitable mechanism to dynamically adjust the receptive fields to match ongoing tasks.

## CONCLUSION

Unlike motoneurons during scratching (Alaburda et al., 2005; Berg et al., 2007), our findings in an integrated spinal cord-hindlimbs preparation show that synaptic activity elicited by natural stimulation in DHN does not override intrinsic membrane properties. Thus, whereas intrinsic properties do not seem to contribute to behaviors that require massive activity of large-scale spinal circuits (Berg and Hounsgaard, 2009), for tasks involving a small number of cells, such as the detection and coding of sensory information, spinal networks may function as those in invertebrates, where intrinsic conductances are main determinants for network output (Destexhe and Marder, 2004).

## AUTHOR CONTRIBUTIONS

The study was done in the department of Neurofisiología Celular y Molecular, Instituto de Investigaciones Biológicas Clemente Estable. Cecilia Reali: conception and design of the experiments; collection, analysis, and interpretation of data; writing of the manuscript; Raúl E. Russo: conception and design of the experiments, collection, analysis, and interpretation of data, writing of the manuscript. All authors approved the final version of the manuscript and are accountable for all aspects of the work.

## Conflict of Interest Statement

The authors declare that the research was conducted in the absence of any commercial or financial relationships that could be construed as a potential conflict of interest.
